# Natural and engineered xylosyl products from microbial source

**DOI:** 10.1007/s13659-024-00435-1

**Published:** 2024-02-01

**Authors:** Jianzhao Qi, Shi-jie Kang, Ling Zhao, Jin‑ming Gao, Chengwei Liu

**Affiliations:** 1 Shaanxi Key Laboratory of Natural Products & Chemical Biology, College of Chemistry & Pharmacy, Northwest A&F University, Yangling, 712100 China; 2 Department of Pharmacy, School of Medicine, Xi’an International University, Xi’an, 710077 China; 3 Key Laboratory for Enzyme and Enzyme‑Like Material Engineering of Heilongjiang, College of Life Science, Northeast Forestry University, Harbin, 150040 China

**Keywords:** Xylosyl product, Cyathane diterpene, Engineering transformation, Xylosyltransferase

## Abstract

Glycosylation is a prevalent post-modification found in natural products and has a significant impact on the structural diversity and activity variation of natural products. Glucosylation is the most common type of glycosylation, whereas xylosylation is relatively rare. Despite their unique chemical structures and beneficial activities, xylosylated natural products from microorganisms have received little attention. This review provides, for the first time, a comprehensive summary of 126 microbial-derived xylosylated natural products, including xylosyl-cyathane diterpenes, xylosylated triterpenes, xylosyl aromatic compounds, and others. Among these compounds, xylosyl-cyathane diterpenes represent the highest number of derivatives, followed by xylosylated triterpenes. Xylosyl compounds from bacterial sources have less defined structural profiles compared to those from fungi. The characterization of xylosyltransferase EriJ from Basidiomycota extended the structural diversity of xylosyl cyathane diterpenes. This work provides a valuable reference for the research and use of xylosyltransferase for drug discovery and synthetic chemistry. Further work is needed to explore the potential applications of microbial derived xylosyl compounds and to develop novel xylosyl transferases. With the deepening of genomic sequencing of medicinal fungi, more biosynthesis of bioactive xylosyl compounds is expected to be elucidated in the future.

**Graphical Abstract:**

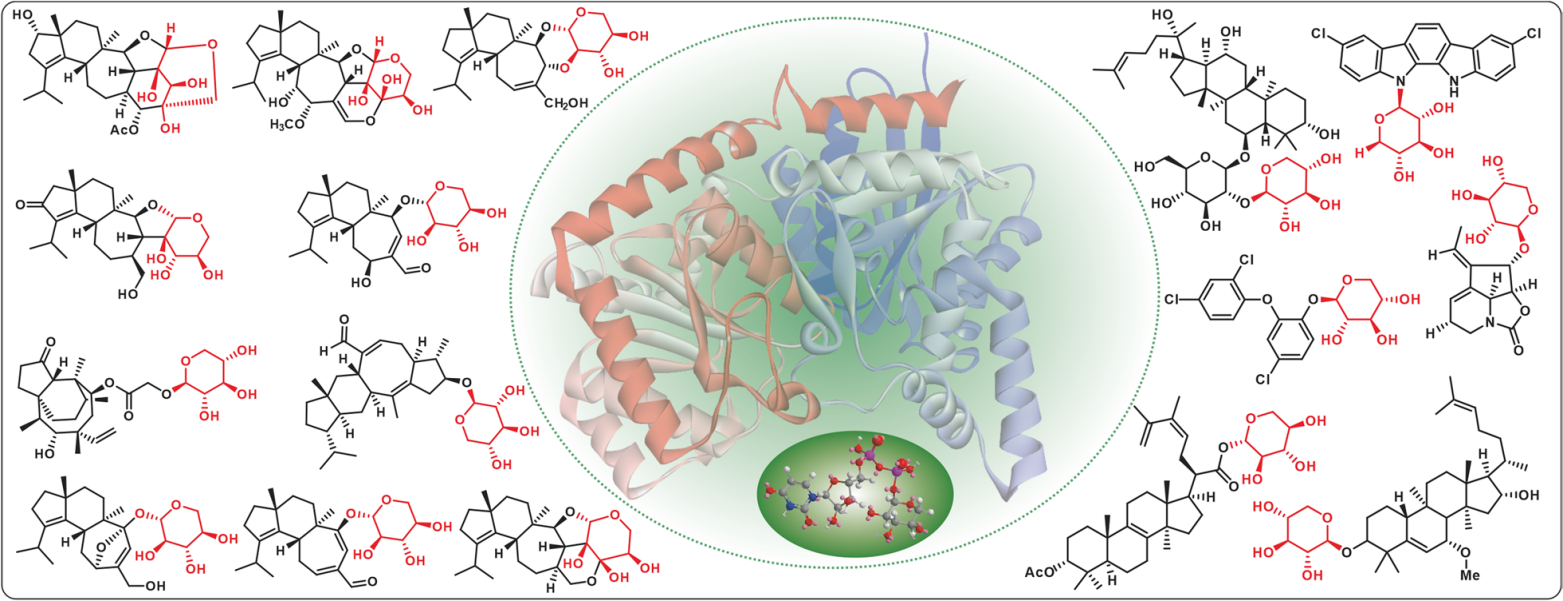

**Supplementary Information:**

The online version contains supplementary material available at 10.1007/s13659-024-00435-1.

## Introduction

Post-modification is a crucial step in the biosynthesis of natural products, which contributes to the formation of structurally diverse compounds with identical scaffolds. These minute structural variations engender differences in their biological activities. Among the various types of post-modifications, glycosylation holds immense importance and uniqueness. Glycosylated natural products, such as ginsenosides [1], are commonly used as bioactive constituents in herbal medicines [2]. It is estimates that natural glycosides make up approximately 17% of the total amount of natural products [3]. Glycosylation modification enhances the diversity of natural product structures and pharmacological activities and influences the physicochemical characteristics of compounds, impacting their pharmacokinetic profiles. Therefore, glycosylation is a widely used approach to derive lead compounds in drug discovery. It is noteworthy that about 5% of approved drugs available on the market are glycosides or oligosaccharides [4].

There is a wide variety of natural monosaccharides, with glucose being the most common. The diversity of monosaccharide types determines the diversity of sugar-based modifications in natural products. Among glycosylated natural products derived from plants [5] or microorganisms [6, 7], glucose modification is the most common type of glycosylation. In addition, xylose modification is an important type of glycosylation, with extensive research focused on triterpene xylosides in Chinese medicinal plants of the *Astragalus* genus [8]. In addition to the above studies, an onoceranoid xyloside with antioxidant activity has been discovered in the peel of *Lansium parasiticum* [9] and rare flavonoid xylosides have been found in plants of the genus *Syzygium* [10].

Microbial-derived xylosyl natural products have received less attention in the natural product chemistry literatures than plant-derived xylosyl natural products. However, microbial-derived xylosyl natural products have unique chemical structures and exhibit good biological activities. For example, triterpene xylosides have anti-inflammatory activity [11] and cyathane diterpene xylosides have anti-neurodegenerative activity [12]. Although their presence is scattered, it is important to recognize their importance and summarise their properties. Since the first report of xylosyl aminoglycoside antibiotics in 1972 [13], numerous microbial-derived xylosyl natural products have been discovered in the last 51 years. This work presents a systematic summary of the producing organisms, structural features and biological activities of 126 natural and engineered xylosyl products. The review enhances our understanding of the structural diversity of these compounds and provides a useful reference for the study of microbial xylosyl transferases.

## Diverse chemical structures and biological activities

### Xyloside triterpenoids

*Hebeloma vinosophyllum* is a poisonous mushroom found in Japan. Fujimoto Haruhiro et al. isolated a series of toxic triterpene glycosides, called hebevinosides, from this mushroom between 1986 and 1991 [1–3]. Among these compounds, components I, III, IV, VI, VII, IX, X, XI, and XII (**1**–**9**), a total of 9 compounds, are xyloside triterpenes, where the xylose moieties are attached to the hydroxyl group of lanosterol at position C3 [14–16]. Compounds **1**–**8** were obtained from the fruiting bodies of *H. vinosophyllum* [14, 15], while **9** was obtained from its mycelial culture [16]. Compounds **1**, **2** were identified as the major toxic components produced by this fungus, and **2** and **4**–**6** were considered to be the major components of these metabolites [14, 15]. The results of toxicity tests conducted on mice indicate that substituting the hydroxyl group at position 7 (**2**, **4**, **5**, **9**) of the compound with methoxy groups (**1**, **3**, **7**, **8**) increases toxicity, while the presence of a glucose moiety at position 16 (**1**, **2**, **4**, **5**, **7**–**9**) is necessary for toxicity to occur [15]. Two new xyloside triterpenoids, tsugariosides B and C (**10**, **11**), were isolated from the methanol extract of the fruiting bodies of *Ganoderma tsugae*, a classical medicinal mushroom. In vitro cytotoxic activity tests were performed on PLC/PRF/5, T-24, 212, HT-3, SiHa, and CaSKi cells, and the results showed that **11** had an ED_50_ value ranging from 6.5 to 9.5 µg/mL [17]. A new xyloside triterpene, Laetiposide E (**12**), was isolated from the ethanol extract of the fruiting bodies of *Laetiporus versisporus*. However, no activity was observed when its inhibitory activity against KB and L1210 cells (ID_50_ > 50 µg/mL) was tested [18].

**Fig. 1:**
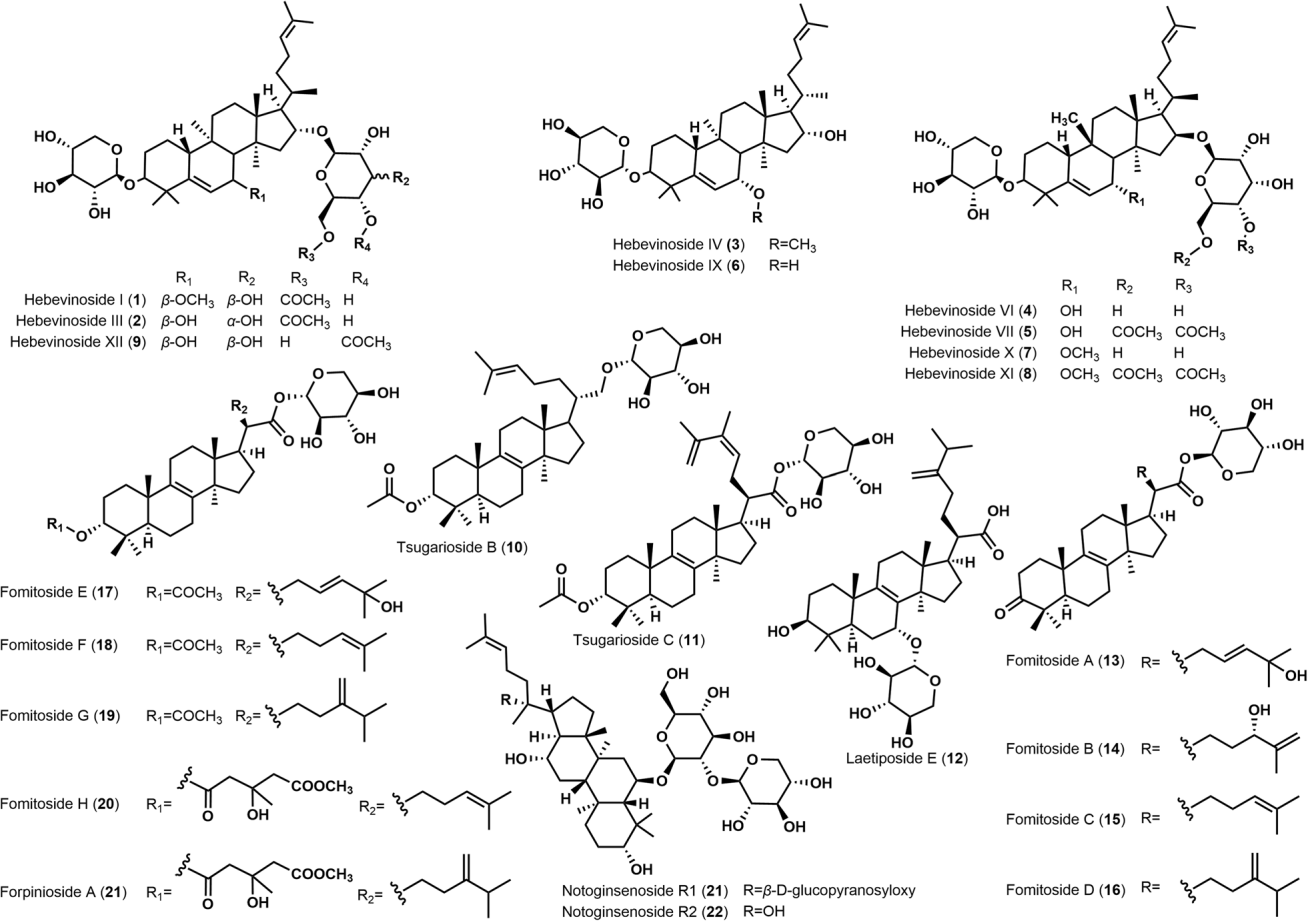
Chemical structures of xylosyl-modified triterpenoids

Eight new xyloside triterpenes, fomitosides A–H (**13**–**20**), were isolated from the 70% ethanol extract of the fruiting bodies of *Fomitopsis pinicola*. Anti-inflammatory activity assays revealed that **17** and **18** exhibited significant inhibitory activity against COX-2 with IC_50_ values of 0.15 µM and 1.13 µM, respectively [11]. This is the first report on the inhibitory activity of lanostane triterpene xylosides on COX-2. Subsequently, a new xyloside lanostane triterpene, forpinioside A (**21**), along with the previously reported **15** and **20** were isolated from the 95% MeOH/H_2_O extract of *F. pinicola* (Sw. Ex Fr.) Krast [19]. The cytotoxic activity of these compounds against five human tumor cell lines, HL-60, A549, SMMC-7721, MCF-7 and SW480, was evaluated by MTS method. The results showed that **21** exhibited relatively good activity against all five cell lines, with IC_50_ values ranging from 11.42 ± 0.39 to 21.06 ± 0.76 µM. In addition, these compounds were tested for their induction of apoptosis in HL-60 cells, and **15** showed weak activity, increasing the percentage of apoptotic HL-60 cells by 8.8% at a concentration of 20 µM [19]. In 2021, Li et al. designed and constructed a yeast chassis strain for the production of protopanaxatriol, and then introduced plant-derived glycosyltransferase-encoding genes (PgUGT71A53 and PgUGT81A54) and xylosyltransferase-encoding gene (PgUGT94Q13) into the chassis, and obtained two triterpenoids containing xylosides Notoginsenoside R1 (**22**) and Notoginsenoside R2 (**23**). This work realized the first microbial production of plant triterpenes **22** and **23** [20]. The chemical structures of these xylosyl triterpenes are displayed in Fig. 1.

### Xyloside cyathane diterpenoids

Cyathane diterpenes are the major type of diterpenes derived from Basidiomycota. These compounds possess a distinct tricyclic ring system comprised of five, six, and seven carbon atoms. The cyathane diterpene scaffold can undergo several types of post-modifications, but xylosylation is the predominant form. Xylosylation specifically occurs specifically at the hydroxyl group located at position C14 on the cyathane diterpene scaffold. The hydroxyl group of xyloses then undergoes further fusion with the cyathane diterpene scaffold, resulting in the formation of highly oxidized polycyclic complex compounds. Naturally occurring xyloside cyathane diterpenoids are commonly found in the metabolites of the genus *Cyathus* [12], *Hericium* [21–23], and two specific mushrooms, *Laxitextum incrustatum* [24] and *Dentipellis fragilis* [25].

#### Xylosyl-cyathane diterpenes from the genus *cyathus*

From the 1970s to the beginning of this century, researchers from Canada, Germany, China and other countries have studied and reported 13 structurally complex cyathane diterpene xylosides produced by *C. striatus*. In 1977, Anke et al. were the first to isolate and obtain three new cyathane diterpenes from *C. striatus*. These three compounds were named as striatins A–C (**24**–**26**) [26]. Compounds **24**–**26** showed broad-spectrum antimicrobial activity, which was particularly significant against *Bacillus subtilis* and *Proteus vulgaris* with MIC values of 0.2–2 µg/mL [26]. Subsequently, three new striatals A–C (**27**–**29**) with keto-aldehyde moieties were isolated and purified from *C. striatus* [27]. In the course of elucidating the biosynthetic pathway of **27**, striatal D (**30**) [28] was discovered, a compound initially isolated from the culture medium of *Gerronema fibula* [28]. In 2014, these four compounds, **27**–**30**, were simultaneously isolated together from *C. striatus* [29]. Both striatins (**24**–**26**) and striatals (**27**–**30**) had antibacterial, antifungal and antileishmanial activities [28]. In addition, they showed antitumor activity [28]. Six new highly oxygenated polycyclic cyathane diterpene xylosides, striatoids A–F (**31**–**36**), were isolated from *C. striatus* in 2015, and it is noteworthy that among them, **32** and **33** possessed structures with unusual 15,4′-ether ring systems [30]. Neurotrophic activity assays showed that **31**–**36** at concentrations of 10–40 µM significantly promoted neurite outgrowth in PC-12 cells under NGF-induced conditions compared to NGF (20 ng/mL) as a positive control [30].

In 2018, researchers in Thailand isolated six new polycyclic cyathane diterpene xylosides cyathinins A–E (**37**–**4**1) and 10-hydroxyerinacine S (**42**) from a new *Cyathus* species, *C. subglobisporus* BCC44381 [31], as well as **26**, **27**, **29**, **30**, and **33**, five compounds previously derived from *C. striatus*. The results of antimicrobial activity tests on compounds other than **38** showed various antimicrobial activities [31]. Compounds **26**, **27**, **29**, **30**, **33**, **37**, and **40** showed antimalarial activity with IC_50_ ranging from 0.88 to 7.51 µM. Compounds **26**, **27**, **29**, and **40** showed *Candida albicans* inhibitory activity with IC_50_ ranging from 8.6 to 80.3 µM. Compounds **26**, **27**, **29**, **37** and **40** showed anti-tuberculosis activity with MIC values ranging from 25.0 to 50.0 µg/mL. Compounds **26**, **27**, **29**, **30**, **37** and **40** showed antibacterial activity against Gram-positive bacteria with MIC values in the range of 0.78–50.0 µg/mL. Compounds **26**, **27** and **29** showed phenylalanine–arginine–β-naphthylamine (PAβN)-dependent inhibitory activity against three Gram-negative bacteria, *Escherichia coli*, *Acinetobacter baumannii*, and *Klebsiella pneumoniae*, with MIC values in the range of 3.13–50 µg/mL. Compounds **33**, **37**, **40** showed PAβN-dependent inhibitory activity against *E. coli* and *A. baumannii* with MIC values in the range of 6.25–50.0 µg/mL [31]. Structure activity relationship analysis exhibited that the hydroxyl group at C-10 might contribute to the antimicrobial activity, and the xylose moiety also had some antimicrobial effect [31]. Me-dentifragilin A (**43**) is a xylose-ornithine recently isolated from the rice fermentation of *C. striatus* CBPFE A06, which exhibits favorable neuroprotective and anti-neuroinflammatory activities [32].

#### Xylosyl-cyathane diterpenes from the genus *Hericium*

*Hericium* mushrooms are recognized as a major source of cyathane diterpenes, with the largest proportion of these diterpenoids being referred to as “erinacines,” which are primarily present in the form of xylose modifications. Erinacines A–C (**44**–**46**) were first isolated and identified as new cyathane diterpene xylosides from the mycelial fermentation broth of *H. erinaceum* by Kawagishi et al. in 1994 [33]. These compounds were found to stimulate the production of NGF in rat brain astrocyte cells [33]. More cyathane diterpene xylosides were extracted from the mycelial fermentation broth of *H. erinaceum* during 1996–2006, with the following compounds named as erinacines D–H (**47**–**51**), and J–K (**52**, **53**) [34–37]. These compounds were also found to stimulate NGF synthesis in rat astrocyte cells [34–37]. The result of anti-methicillin-resistant *Staphylococcus aureus* (MRSA) assay indicated that **44**, **46**, and **53** possessed substantial anti-MRSA activities with MIC values ranging between 62.5 and 500 µM, while **52**, with 3,4-seco-scalffold, did not exhibit any such activity [37]. Consequently, it was hypothesized that the tricyclic scaffold is vital for anti-MRSA properties.

Two novel cyathane diterpene xylosides (**54**, **55**), in addition to a previously reported compound **47**, were isolated from the mycelial fermentation broth of *H. erinaceum* as report by Atsushi et al. in 1996 [38]. Saito et al. reported in 1998 the extraction and isolation of **48** from the liquid fermentation broth of uncommon *Hericium* species, *H. ramosum* CL24240 [39], and they found two new compounds, CJ-14258 (**56**) and CJ-15544 (**57**), by altering the conditions of the fermentation broth. Using *Cladosporium fumago* ATCC 16373 as the chassis, **48** was biotransformed into a new compound CP-412065 (**58**) [39]. It was found that **48**, **56**, **57** could inhibit Kappa-opioid receptors [39]. In the years 2000–2002, Kenmoku et al. isolated erinacines P and Q (**59**, **60**) from the fermentation broth of *H. erinaceus* YB4-6237 [40, 41]. Compound **59** was observed to undergo biomimetic transformation to yield **45** under mild conditions, which in turn could be further transformed to **44** [29]. From a biosynthetic perspective, **60** appeared to serve as the precursor of compound **59**. Additionally, compound **60** might act as the parent metabolite of **44**–**46**. In feeding experiments with [1′-^13^C]**-59** and [1′-^13^C]**-60**, it was evident that in *H. erinaceus* YB4-6237, **60** was converted to **46** through **59**. It is probable that **59** and **60** are common biosynthetic intermediates that *H. erinaceus* employs to produce cyathane xylosides [41].

Erinacine R (**61**) was a cyathane diterpene xyloside that was extracted from the mycelium of *H. erinaceum* using methanol. Its relative stereo structure was determined through ROESY [42]. Compound **62**, another cyathane diterpene xyloside, was also extracted from the mycelium of *H. erinaceum* and its absolute configuration was determined using vibrational circular dichroism (VCD) and calculated VCD due to its antimicrobial activity. Compound **62** displayed effective growth inhibition against *Helicobacter pylori* ATCC43504 with MIC values ranging between 50 and 100 µM. Additionally, Compound **62** demonstrated good cytotoxicity against the human erythroleukemia cell line K562 and human laryngeal epithelial cell line HEP2 with IC_50_ less than 200 mM [43]. Erinacine S (**63**) is a new xyloside guanosine isolated from the ethanolic extract of the mycelium of *H. erinaceum*. It has been shown that **63** may increase the degradation of Aβ amyloid by increasing IDE levels, thereby reducing the burden of AB10-stained plaques [44].

In 2018, Zhang et al. isolated three novel erinacines (T–V, **64**–**66**) along with the previously identified compounds **44** and **59** from the liquid medium of *H. erinaceum*. Their structures were determined using a comprehensive spectral analysis. Of the five compounds evaluated, only **44** exhibited weak cytotoxicity against PC12 cells, with an IC_50_ of 73.7 µM. Compounds **64**–**66** and **59** produced a significant decrease in the range of 2.5–10 µM, indicating significant neurotrophic effects when compared to the NGF control [45]. In 2018, Rupcic et al. identified eight cyathane diterpene xylosides from the mycelium of *H. erinaceum*, and a rare species *H. flagellum.* These cyathane diterpene xylosides included erinacines Z2 (**64**) and Z1 (**65**), as well as six previously reported erinacines **44**–**46**, **48**, **49**, and **56** [46]. Two newly identified erinacines, Z2 (**64**) and Z1 (**65**), have the same structure as previously reported erinacines T (**64**) and U (**65**), which were previously reported [45]. These eight compounds were tested for differentiation activity in promoting neuronal growth in PC12 cells, with weaker activity of erinacines **64**, **65** and stronger activity of **44**–**46**, **48**, **49**, and **56** [46].

In the same year, three previously undescribed cyathane diterpene xylosides, newly named hericinoids A–C (**67**, **43**, **68**), as well as three already reported erinacines, **64**, **65**, and **57** were reported to have been isolated from the fermentation broth of *H. erinaceum*. Among them, the absolute conformations of **67** and **43** were determined by ROESY correlation and DP4+ calculations. None of these five compounds showed promoting effects on NGF-induced neurite growth in PC-12 cells. Cytotoxicity assays showed that **43**, **64**, **65** displayed significant cytotoxicity against HL-60 cell line with IC_50_ values of 18.3, 8.9 and 0.5 µM, respectively, and **64**, **65** exhibited moderate cytotoxicity against the MCF-7 cell line with IC_50_ values of 13.4–15.8 µM [47]. Erinacine L (**69**) is a recently reported xylose-cyathane with a rare hemiacetal moiety isolated from the rice medium of *H. erinaceus* CGMCC 5.579, which showed a significant inhibitory effect on NO synthases involved in neuroinflammatory pathways, with IC_50_ values as low as 5.82 µM [48].

#### Xylosyl-cyathane diterpenes from *L. incrustatum*

In 2016, Mudalungu et al. isolated two new cyathane diterpene xylosides, laxitextines A and B (**70**, **71**), as well as the previously reported **30**, from mycelial extracts of *L. incrustatum* [24]. The inhibitory concentrations of **70**, **71** against *B. subtilis* DSM 10 were 33.3 µg/mL (76.7 µM) and 16.7 µg/mL (37.4 µM), respectively. Compound **70** also showed significant inhibitory activity against *S. aureus* DSM 346 and MRSA DSM 1182 with a MIC of 7.8 µg/mL (17.9 µM). However, **71** showed weaker anti-MRSA activity with a MIC of 62.5 µg/mL (140.0 µM). Compounds **70**, **71** showed moderate activity against the mouse fibroblast cell line L929 and the human mammary carcinoma MCF-7, epidermoid carcinoma A431, and umbilical vein endothelial cells. Among these, the strongest inhibitory activity was observed against the MCF-7 cell line with IC_50_ values of 2.3 and 2.0 µM, respectively [24].

#### Xylosyl-cyathane diterpenes from *D. fragilis*

The erinacines A–C (**44**–**46**) are known metabolites of *Hericium* mushrooms, but they were isolated from the fermentation broth of *D. fragilis* in 2021. The results of antimicrobial activity assays showed that **45**, **46** exhibited relatively effective antimicrobial activity against *B. atrophaeus*, *S. epidermidis*, and *B. subtilis* with MIC values ranging from 2.5 to 10 µg/mL, and they showed strong antifungal activity against *B. cinerea*, *C. demantium*, and *F. oxysporum*, with MIC values ranging from 10 to 20 µg/disc [49]. This is the first report of **44**–**46** being isolated from mushrooms other than those of the genus *Hericium*. Subsequently, eight unreported cyathane diterpene xylosides, dentifragilins A–H (**72**–**79**), as well as two previously reported **30** and **70**, were isolated from the submerged fermentation broth of *D. fragilis*. Compound **72** was found to have potent antimicrobial activity with MICs of 1.0 µg/mL against *B. subtilis* and 4.2 µg/mL against *S. aureus*, whereas **75**–**76** had moderate antimicrobial activity with MICs of 16.4–33.3 µg/mL against *B. subtilis* and *S. aureus*. Cytotoxicity tests showed that **30**, **72**, and **79** exhibited significant activity. Among them, **30** showed the strongest cytotoxic activity against the ovarian cancer cell line SKOV-3, squamous cell carcinoma A549, human breast cancer cell MCF-7, mouse fibroblast l929 cells, and human endocervical adenocarcinoma cell lines KB3.1, with IC_50_ values not exceeding 0.8 µM [25].

#### Xylosyl-cyathane diterpenes produced by engineered producers

Heterologous expression of the biosynthetic gene cluster (BGC) for erinacines by *Aspergillus oryzae* chassis led to the first efficient heterologous production of **60**, as well as erinacine Q2 (**60b**), a non-natural glucose-cyathane diterpene. Enzymatic reactions showed that the glycosyltransferase encoded by *eriJ* catalyzed the production of erinacine Q (**60**) from **60a** using UDP-Xyl as the glycosyl donor, while EriJ could also catalyze the production of erinacine Q2 (**60b**) from 60a using UDP-Glu as the glycosyl donor. EriJ was the first xylosyltransferase to be characterized in Basidiomycota [50].

**Fig. 2:**
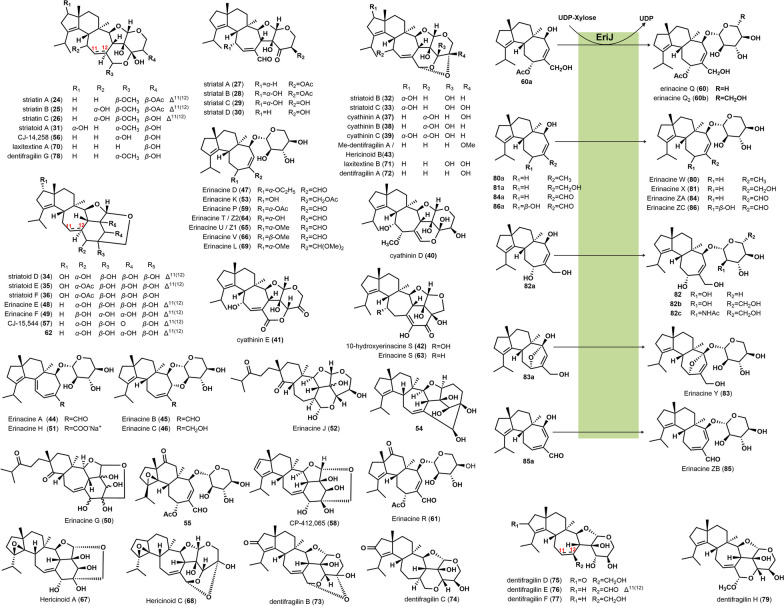
Chemical structures of xylosyl-modified cyathane diterpenoids and their derivatives

Three novel cyathane diterpene xylosides, named erinacines W, X (**80**, **81**), and an unnamed compound **82**, were synthesized in *Saccharomyces cerevisiae* by substrate feeding. These compounds showed nerve growth factor (NGF)-dependent neurotrophic activity. Administration of these compounds to mice in the Morris water maze test showed significant improvements in cognitive performance at doses of 5 mg/kg and 10 mg/kg [51]. In addition, six naturally occurring erinacines (**44**–**46**, **59**, **60**, **64**) and seven unnatural cyathane diterpene xylosides, including **80**–**82**, erinacine Y (**83**), and erinacines ZA-ZC (**84**–**86**), were produced in genetically engineered *S. cerevisiae* using a combinatorial biosynthetic approach. During enzymatic reactions in vitro, the enzyme EriJ performed xylosylation on different hydroxylated cyathane backbones (**80a**–**83a**), resulting in compounds **80**–**83**. EriJ was also found to use UDP-glucose and UDP-*N*-acetylglucosamine as glycosyl donors to catalyze the synthesis of cyathane diterpene glucoside (**82b**) and cyathane diterpene *N*-acetyl-glucoside (**82c**), respectively [52]. Assessment of pro-neuronal growth activity using PC12 cells showed that **80**–**84** exhibited substantial neurotrophic effects at concentrations ranging from 6.3 to 25.0 µM. Compounds **81** and **82** showed higher activity than **80**. Preliminary SAR analysis suggested that hydroxylation at the C11 and C15 positions on the cyathane scaffold enhances neurotrophic effects [52]. The chemical structures of the above xylosyl cyathane diterpenes and their derivatives are displayed in Fig. 2.

### Xylosyl aromatic compounds

Benamomicins A–B (**87**, **88**) are xylosylated antibiotics with a benzo[α]naphthacene quinone scaffold, which were discovered in the culture broth of *Actinomycete* sp. MH193-16F4 in 1988 [53]. **87**, **88** exhibited inhibitory activity against fungi and gram-positive bacteria, with MIC values of 3.13 µg/mL and 1.56 µg/mL against *Cryptococcus neoformans* F-10, respectively [53]. Subsequently, the anti-AIDS activity of **87** and **88** was reported [54]. They inhibited de novo infection of human T-cells with HIV-1 and also inhibited syncytium formation of human T-cells after cocultivation with HIV-1-producing cells [54]. Later, a new benamomicin named 2′-demethylbenanomicin A (**89**) was discovered in the culture broth of *Actinomadura* sp. MH193-16F4, which is a product of methylation removal from the phenyl ring side chain of **87**. Compound **89** had similar antifungal activity with **87** [55].

BMY-28567 (**90**) was isolated from the supernatant of the fermentation of *A. hibisca* No. P157-2 (ATCC 53557) [56]. Compound **90** had a broader antibacterial spectrum, not only inhibiting *Candida*, *Aspergillus*, *Microsporum*, *Penicillium* and *Sporothrix* with 5-fluorocytosine and amphotericin B resistance, but also inhibiting Gram-positive bacteria such as *Micrococcus luteus* (MIC: 3.1 µg/mL), *Mycobacterium* species (MIC: 12.5–25.0 µg/mL) [56]. It is worth mentioning that **90** showed good therapeutic effect on systemic infections caused by *Candida albicans* (PD_50_: 4.5–11.0 mg/kg) and *Cryptococcus neoformans* (PD_50_: 16–35 mg/kg). In addition, **90** inhibited the growth of influenza A virus in MDCK cells (ID_50_: 6.8 µg/mL; TD_50_: > 200 µg/mL) and also could activate host defense in *Pseudomonas aeruginosa*-infected mice [56].

In 1989, pradimicin A (**90**) and pradimicin C (**88**) were isolated from the culture broth of *A. hibisca* P157-2 (ATCC 53557) [57]. Interestingly, the structure of pradimicin A is identical to BMY-28567 (**90**), while pradimicin C has the same structure as benamomicin B (**88**). Compound **90** exhibited MIC values of 0.8–12.5 µg/mL against various fungi and yeasts in vitro, and an EC_50_ value of 0.33% against *vaginal candidiasis*. Compound **88** showed similar antifungal activity with MIC values of 0.8–3.1 µg/mL against various fungi and yeasts, but its antibacterial spectrum is slightly lower than that of **90** [58]. At concentrations exceeding 3.5 µg/mL, **90** inhibited HIV-induced cell damage and showed anti-HIV activity during virus attachment and cell-to-cell infection stages [59].

Further studies on cultures of *A. hibisca* P157-2 identified two new pradimicins, pradimicin D (**91**) and pradimicin E (**92**) [60]. They are glycine analogs of Pradimicin A (**90**) and Pradimicin C (**88**). Due to the low levels of compounds **91** and **92** in this strain, the strain was treated by *N*-methyl-*N*′-nitro-*N*′-nitrosoguanidine (MNNG) to obtain the mutant strain *A. hibisca* No. A2660 (ATCC 53762). This mutant was able to produce **91** and **92** in large quantities [60]. The structures of **91** and **92** were determined by comparative analysis with the comprehensive spectra of **90**. **91** and **92** displayed a similar antifungal activity to **90** with MIC values ranging from 0.8 to 6.3 µg/mL and 0.8–12.5 µg/mL respectively against most fungi and yeasts, but showed no significant inhibition against *P. boydii*. In terms of systemic antifungal activity against *Candidiasis* in mice, the PD_50_ values of **91** and **92** were 9.0 mg/kg and 8.9 mg/kg, respectively, which were comparable to the activity of **90** [60]. Adopting the concept of directed biosynthesis, the addition of d-serine to the culture medium of *A. hibisca* P157-2 and its mutant strain *A. hibisca* A2493 led to the production of two new compounds, Pradimicin FA-1 (**93**) and Pradimicin FA-2 (**94**) [61]. The structures of **93** and **94** were also determined through comparative analysis with the comprehensive spectra of **90**. **93** and **94** exhibited similar in vitro antifungal activity to **90** with MIC values ranging from 0.8 to 12.5 µg/mL and 0.8–6.3 µg/mL against most fungi and yeasts, although **94** showed weaker inhibition against *P. boydii* [61]. In terms of systemic antifungal activity against *Candidiasis* in mice, **93** (PD_50_: 18 mg/kg) exhibited lower activity compared to **90** (PD_50_: 8.9 mg/kg) and **94** (PD_50_: 7.4 mg/kg) [61].

In 1993, Furumai et al. isolated two new pradimicins analogs, pradimicin T1(**95**) and pradimicin T2 (**96**), from the culture broth of *A.* AA3798 [62], which were structurally characterized by l-xylose substitution at the C-11 OH on their backbones. Compound **95** had a broader in vitro antifungal activity compared to **96**, with MIC values ranging from 1.6 to 25 µg/mL against most fungi and yeasts, whereas **96** showed MIC values ranging from 1.6 to 12.5 µg/mL against most fungi and yeasts. However, both **95** and **96** were poorly effective against *A. fumigatus* and *T. mentagrophytes* [63]. Compound **95** (15 mg/kg) was more active against *C. albicans* systemic infection in mice than **96** (54 mg/kg). In addition, **95** had better antiviral activity [63]. In the same year, Furumai et al. obtained the mutant strain JN-380 after *N*-methyl-*N*′-nitro-*N*′-nitrosoguanidine (NTG) induction treatment of *A. verrucosospora* subsp. *neohibisca* R103-3. Two new pradimicins derivatives, pradimicin H (**97**) and pradimicin FH (**98**), were identified from the culture broth of the mutant strain [64]. Another two new 11-*O*-l-xylosyl homologs, 11-*O*-l-xylosylpradimicin H (**99**) and 11-*O*-l-xylosylpradimicin FH (**100**), were subsequently obtained by feeding *A.* A3798 with **97** and **98** as substrates. The in vitro antifungal activities of **99** and **100** were assayed, and the results showed that **99** and **100** possessed a wide range of antifungal activities with MIC values ranging from 1.6 to 25 µg/mL, while no cross-resistance with amphotericin B or ketoconazole. Compounds **99** and **100** had in vivo anti-infective against *C. albicans* A9540 in mice with PD_50_ values of 18 mg/kg and 20 mg/kg, respectively, and had no acute toxicity (LD_50_ > 300 mg/kg) [64] .

**Fig. 3:**
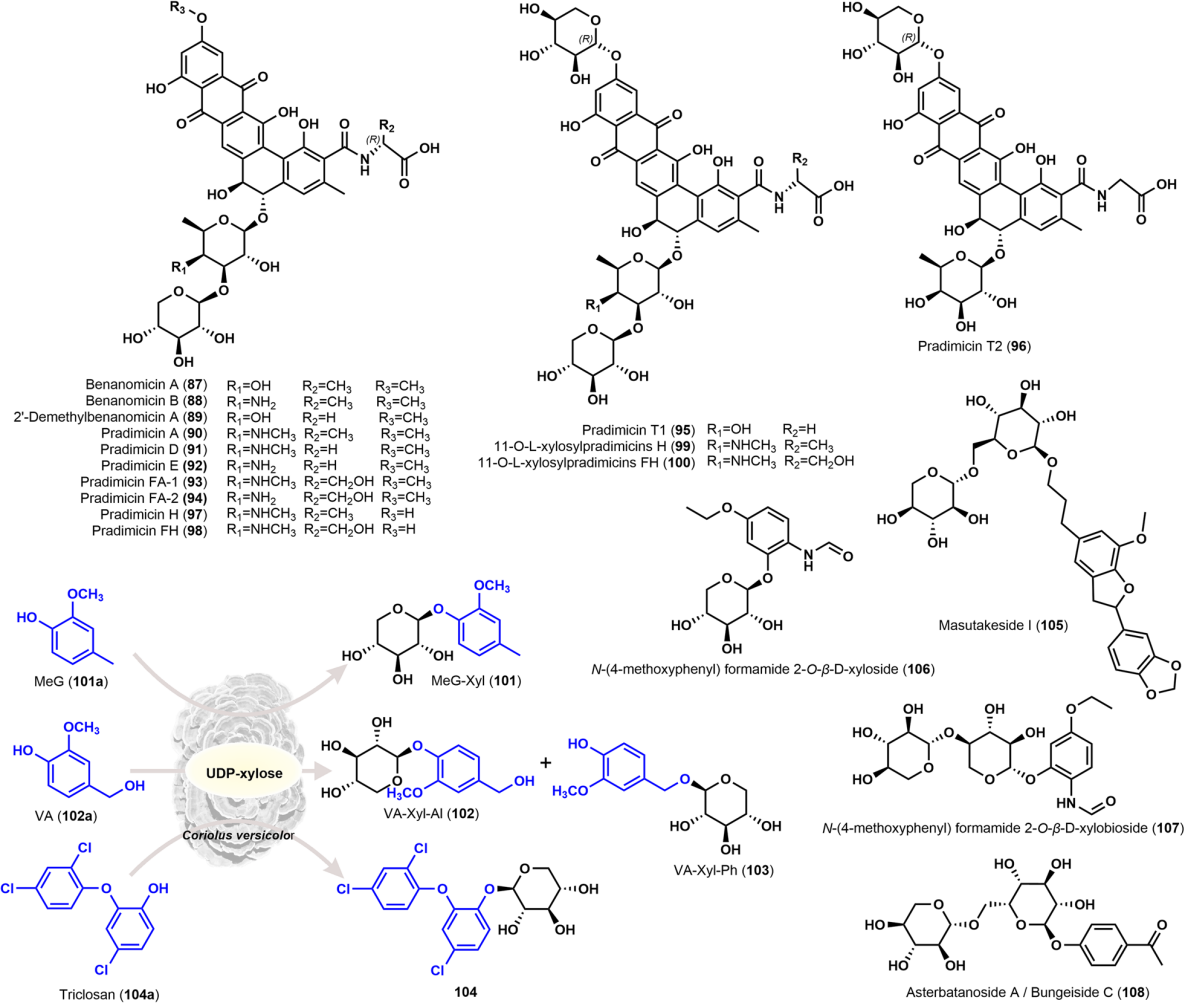
Chemical structures of xylosyl-modified aromatic compounds

4-Methylguaiacol (MeG) and vanillyl alcohol (VA) were added to the medium of *Coriolus versicolor* for transformation culture, respectively. The former was transformed into 2-methoxy-4-methylphenyl *β*-d-xyloside (MeG-Xyl, **101**), while the latter was transformed to form vanillyl *β*-d-xyloside (VA-Xyl-Al, **102**) and 2 methoxy-4-hydroxymethylphenyl *β*-d-xyloside (VA-Xyl-Ph, **103**). This result implied that the phenolic hydroxyl groups in VA were more susceptible to xylosylation than the alcoholic hydroxyl groups [65]. Thereafter, the xylosylation of triclosan (**104a**) was obtained by adding **104a** to the medium of *Trametes versicolor*, while co-incubation of cell extracts of *T. versicolor* with **104a** and UDP-xylose resulted to yielding **104** [66]. This study also found that the cytotoxicity of **104** was lower than that of **104a** itself, which implied that xylosyl modification of exogenous substances might be a detoxification mechanism for the fungus. A new benzofuran xyloside, masutakeside I (**105**), was isolated from the ethanolic extract of the fruiting body of *Laetiporus sulphureus* var. *miniatus*, which showed no significant toxicity to Kato III cells [67]. Two new aromatic xylosides, *N*-(4-methoxyphenyl) formamide 2-*O*-*β*-d-xyloside (**106**) and *N*-(4-methoxyphenyl) formamide 2-*O*-*β*-d-xylobioside (**107**). It was hypothesized that low concentrations of **106** might possess the function of enhancing the viability of BEAS-2B cells [68]. An aromatic glycosylated compound asterbatanoside A, also known as bungeiside C (**108**) was isolated from the ethyl acetate extract of the endophytic fungus *Plectosphaerella cucumerina* YCTA2Z1 from *Psoralea cucumerina* [69]. This compound was originally isolated from the roots of *Cynanchum bungei* Decne [70], and the present study is the first to report its microbial origin. The chemical structures of these xylosyl triterpenes are displayed in Fig. 3.

### Miscellaneous compounds containing xylose moieties

A-40104 antibiotic complex was the active ingredient isolated from the deep aerated fermentation broth of *Clitopilus pseudo-pinsitus*. The main component of the complex, A-40104 A (**109**), was a xylosylated derivative of the known antibiotic pleuromutilin (A-40104 C) [71]. Compound **109** had a broad spectrum of antibacterial activity, with significant inhibitory activity against anaerobes, and mycoplasmas, especially against gram-positive bacteria, such as *S. aureus* and *Streptococcus faecalis*, with MIC values < 0.5 µg/mL. In addition, **109** has been shown to inhibit the growth of drug-resistant bacteria [71].

Cepacidines A1 (**110**) and A2 (**111**) are glycopeptide antibiotics isolated from the fermentation broth of *Pseudomonas cepacian* AF 2001. The structures of the two are very similar, the only difference being the presence of a β-hydroxyl modification on the asparagine residue of **110** as opposed to **111** [72]. The mixture of the two has a wide range of in vitro antifungal activity and is highly active against most fungi, especially dermatophytes (*M. canis*, *Trichophyton* spp. and *Epidermophyton* spp.) and yeasts at concentrations below 0.049 µg/mL [73]. Aeruginosins 205 A (**112**) and B (**113**) were two new glycopeptides obtained from the lyophilized mycobacterium of the cyanobacterium *Oscillatoria agardhii* NIES-205, and their absolute configurations were determined by comprehensive NMR analysis. Both inhibited not only trypsin, with an IC_50_ of 0.07 µg/mL, but also thrombin, with IC_50_ values of 1.5 µg/mL and 0.17 µg/mL, respectively [74]. Insertional mutagenesis of an NRPS-containing gene cluster into the genome of the cyanobacterium *Planktothrix agardhii* CYA126/8 produced two glycopeptides, aeruginosides 126 A (**114**) and B (**115**) [75].

Occidiofungins A (**116**) and B (**117**) were isolated as two cyclic octapeptide glycopeptide antibiotics from the liquid medium of the bacterium *Burkholderia contaminans* MS14. They differ structurally by the difference in one of the eight amino acids forming the backbone, the former being asparagine and the latter β-hydroxyasparagine [76]. The mixture composed of the two had a broad-spectrum antifungal effect and showed significant antifungal activity against a variety of plant and animal fungal pathogens, especially against *R. solani*, which had a strong inhibitory effect with a MIC of 2 µg/mL. This mixture had MIC values of 8 µg/mL and 4 µg/mL against *A. fumigatus* and *A. niger*, 4 µg/mL against *M. gypseum* and *T. mentagrophytes*, and 1 µg/mL and 2 µg/mL against *P. spinosum* and *P. ultimum*, respectively [76]. The antifungal mechanism of **116** and **117** was thought to exert antimicrobial effects by disrupting cell wall formation [76]. Bk-1229 (**118**) was a new lipopeptide obtained from spray-dried cells of the bacterium *(B) ambifaria* 2.2 N, and its absolute configuration was determined by comprehensive NMR analysis. The antimicrobial activity assay showed that **118** had a MIC of 0.4 µg/mL against *Saccharomyces cerevisiae* and a MIC of 12.5 µg/mL against *(C) albicans* and *A. niger* [77].

Butirosin A (**119**), a rare xylosyl-modified aminoglycoside antibiotic, was isolated from the fermentation broth of *Bacillus circulans*. In vitro and in vivo antimicrobial tests showed that it exhibited significant activity against many gram-positive and some gram-negative bacteria [13]. Since then, the xylosyl-modified antibiotic Xylostasin (**120**) was isolated from the fermentation broth of *Bacillus* sp. Y-399 and *Bacillus *sp. V-7 [78]. Structurally, it is a degradation product of Butirosin A (**119**). Compound **120** showed some inhibitory activity against gram-positive and some Gram-negative bacteria, and its MIC value against Klebsiella pneumoniae was 0.78 µg/mL [78]. Tjipanazole B (**121**), tjipanazole F1 (**122**), and tjipanazole F2 (**123**) were isolated from *Tolypothrix tjipanasensis* as three xylosylated indole alkaloids. Unfortunately, they did not show any significant biological activity [79]. 5-*O*-(*β*-d-Xylopyranosyl) streptazolin (**124**) was a new xylosylated alkaloid isolated from the fermentation broth of *Streptomyces* sp. strain A1, which exhibited significant cytostatic activity against gastric adenocarcinoma HMO2, hepatocellular carcinoma HePG2, breast cancer MCF7 and colon cancer Kato III with GI50 values ranging from 0.15 to 10 µM [80].

**Fig. 4:**
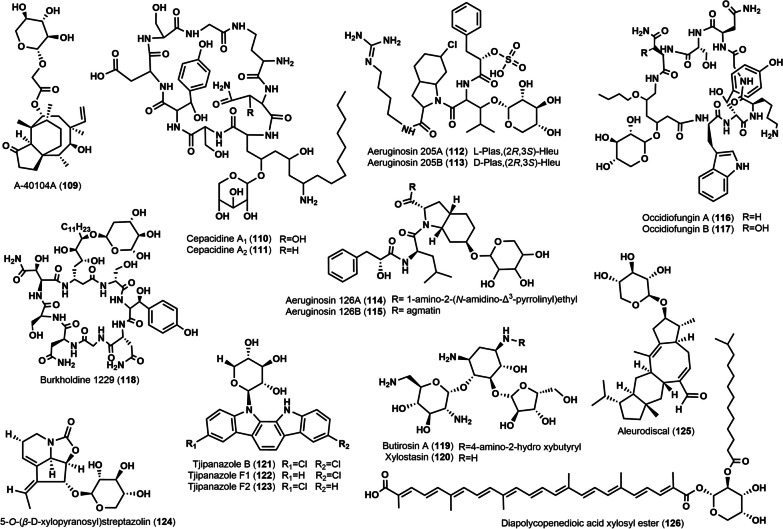
Chemical structures of miscellaneous compounds containing xylose moieties

Aleurodiscal (**125**) was a sesquiterpene isolated from the mycelial fermentation broth of *Aleurodiscus mirabili*s. Plate diffusion assay showed that compound **125** at a concentration of 1 µg/mL inhibited the growth of *Mucor miehei* by 50%, while at a concentration of 10 µg/mL almost completely inhibited the growth of *M. miehei*. The results of cytotoxicity assay showed that **125** at a concentration of 40 µg/mL induced 50% lysis of mouse embryo Balb/3T3 cells and reduced the proliferation of Ehrlich ascites carcinoma cells after 48 h of induction [81]. Diapolycopenedioic acid xylosyl ester (**126**) as a new acyl glyco-carotenoic acid was isolated from the fermentation broth of a marine microorganism, *Rubritalea squalenifaciens*, and its structure was obtained to be characterized by comprehensive MS and NMR analysis. It showed significant in vitro inhibition of free radical-induced lipid peroxidation in rat brain homogenates with an IC_50_ value of 4.6 µM [82]. The chemical structures of these xylosyl triterpenes are displayed in Fig. 4.

## Discussion and perspective

The names, producers, isolated sources and biological activities of all xylosylated compounds are summarised on Additional file 1: Table S1. Out of the 126 microbial-derived compounds, the highest number of xylosylated compounds are found in xylosyl-cyathane diterpenes, with a total of 63, accounting for exactly half. Among these 63 xylosyl-cyathane diterpenes, seven non-natural xylosylated compounds are produced through engineered *S. cerevisiae* [52], while the remaining compounds are derived from Basidiomycota, namely mushrooms. In addition, an antibacterial xylosylated diterpenoids, pleuromutilin compound (**109**), was also found in mushrooms. This compound was discovered as an antibiotic from the *Clitopilus* genus of mushrooms [71]. The group with the second largest number of compounds is that of xylosylated triterpenes, which comprises a total of 23 compounds, all of which are lanostane triterpenes. Among these, notoginsenosides R1 and R2 (22 and 23) are originally produced by *Panax* plants but can also be produced by engineered *S. cerevisiae* [20]. The remaining 21 xylosylated triterpenes are derived from mushrooms. Glycosylated triterpenes are a significant component of mushroom secondary metabolites, dominated by glycosylated triterpenes. Xylosylated triterpenes can be considered by-products of glycosylation modifications. For instance, glycosylated triterpenoids are widespread in the metabolites of the *Ganoderma* genus [83], while only two xylosylated triterpenes (**10**, **11**) have been identified in the *G. tsugae* [17]. Xylosyl aromatic compounds (**87**–**100**) were discovered from actinomycetes in the 1980 to 1990s, towards the end of the antibiotic discovery era [84], and were subsequently prescribed as antibiotics. Unlike the structurally well-characterised xyloside cyathane diterpenes and xyloside triterpenes produced by fungi, the xylosyl compounds from bacterial sources lack a well-defined structural profile.

Glycosylation reactions in biosynthesis are catalyzed by glycosyltransferases, which transfer glycosyl units from an activated glycosyl donor to an acceptor in the formation of region- and stereospecific glycosidic linkages. The most common glycosyl donors are activated nucleotide sugars and phosphate sugars [85], with nucleotide sugars being dominated by uridine diphosphate glucose (UDP-glucose) [86]. Compared to the well-studied glycosyltransferases in plants, glycosyltransferases of microbial origin have received less attention. EriJ is the first xylosyltransferase to be characterized and identified in Basidiomycota [50]. It was found that EriJ catalyzes xylosyl modification of multiple cyathane diterpene scaffold with C14 hydroxyl groups, using UDP-xylose as the glycosyl donor. EriJ is not solely a xylosyltransferase and exhibits broad substrate promiscuity [50]. Heterologous expression in *A. oryzae* chassis showed that EriJ can recognize and utilize UDP-glucose as well as UDP-xylose [50]. Recombinant EriJ protein obtained using *E. coli* also exhibited recognition and utilization of UDP-*N*-acetylglucosamine [52]. The engineering application of EriJ has expanded the structural diversity of xylosyl cyathane diterpenes and provides an excellent example for the mining and application of mushroom-derived xylosyltransferase.

The present study provides a comprehensive compilation and summary of naturally occurring and engineered xylosyl compounds derived from microorganisms. To the best of our knowledge, this is the first-ever summary that outlines the producing organisms, chemical structures, and biological activities of xylosyl compounds from microbial sources. This research not only enhances our understanding of the structural diversity of xylosyl compounds and even glycosyl compounds, but also serves as a valuable reference for the exploration and utilization of xylosyltransferase. With the increasing sequencing of medicinal fungi genomes [87–89], we anticipate that the biosynthesis of more xylosylated active components from medicinal fungi will be unveiled in the future. In summary, further in-depth research is warranted for xylose-based natural products from microbial origins due to their potential applications in drug discovery and synthetic biology. Moving forward, we can expect to see more dedicated research aimed at uncovering the potential uses of these compounds and developing novel xylosyltransferase.

### Supplementary Information

**Additional file 1: Table S1.** The name, bioactivity and source of xylosyl products from microbial source.

## Data Availability

Not applicable.
