# Supplementary material for: Natural and engineered xylosyl products from microbial source
Source: Nat Prod Bioprospect. 2024 Feb 1;14(1):13. doi: 10.1007/s13659-024-00435-1 (PMC10830979; doi:10.1007/s13659-024-00435-1)
Supplement: Supplementary file 1 — Additional file 1: Table S1. The name, bioactivity and source of xylosyl products from microbial source. [file 13659_2024_435_MOESM1_ESM.pdf]

Additional file 1

Natural and Engineered Xylosyl Products from Microbial Source

Jianzhao Qi[123*], Shi-jie Kang[1], Ling Zhao[2], Jin-ming Gao[1] and Chengwei Liu[1,3*]

[1]Shaanxi Key Laboratory of Natural Products & Chemical Biology, College of Chemistry & Pharmacy, Northwest A&F University, Yangling 712100, China.

[2] Department of Pharmacy, School of Medicine, Xi'an International University, Xi'an 710077, China

[3] Key Laboratory for Enzyme and Enzyme-Like Material Engineering of Heilongjiang, College of Life Science, Northeast Forestry University, Harbin 150040, China.

*Correspondence:

Jianzhao Qi: qjz@nwafu.edu.cn, ORCID: 0000-0003-1418-873X

Chengwei Liu: liuchw@nefu.edu.cn, ORCID: 0000-0003-2746-2080

Table S1: The name, bioactivity and source of xylosyl products from microbial source.

NO.	Name	Resource	Isolation site	Activity	Reference
1	Hebevinoside I	*Hebeloma vinosophyllum*	fruiting bodies	Cell toxicity	[1]
2	Hebevinoside III	*H. vinosophyllum*	fruiting bodies	Cell toxicity	[1]
3	Hebevinoside IV	*H. vinosophyllum*	fruiting bodies		[1]
4	Hebevinoside VI	*H. vinosophyllum*	fruiting bodies		[2]
5	Hebevinoside VII	*H. vinosophyllum*	fruiting bodies		[2]
6	Hebevinoside IX	*H. vinosophyllum*	fruiting bodies		[2]
7	Hebevinoside X	*H. vinosophyllum*	fruiting bodies		[2]
8	Hebevinoside XI	*H. vinosophyllum*	fruiting bodies		[2]
9	Hebevinoside XII	*H. vinosophyllum*	Mycelium		[3]
10	Tsugarioside B	*Ganoderma tsugae*	fruiting bodies		[4]
11	Tsugarioside C	*G. tsugae*	fruiting bodies	Antitumor activity	[4]
12	Laetiposide E	*Laetiporus versisporus*	fruiting bodies		[5]
13	Fomitoside A	*Fomitopsis pinicola*	fruiting bodies	Anti-inflammatory activity	[6]
14	Fomitoside B	*F. pinicola*	fruiting bodies	Anti-inflammatory activity	[6]
15	Fomitoside C	*F. pinicola*<br>*F. pinicola* (Sw. Ex Fr.) Krast	fruiting bodies	Anti-inflammatory activity;<br>Antitumor activity	[6, 7]
16	Fomitoside D	*F. pinicola*	fruiting bodies	Anti-inflammatory activity	[6]
17	Fomitoside E	*F. pinicola*	fruiting bodies	Anti-inflammatory activity	[6]
18	Fomitoside F	*F. pinicola*	fruiting bodies	Anti-inflammatory activity	[6]
19	Fomitoside G	*F. pinicola*	fruiting bodies	Anti-inflammatory activity	[6]
20	Fomitoside H	*F. pinicola*<br>*F. pinicola* (Sw. Ex Fr.) Krast	fruiting bodies	Anti-inflammatory activity;<br>Antitumor activity	[6, 7]
21	Forpinioside A	*F. pinicola* (Sw. Ex Fr.) Krast	fruiting bodies	Antitumor activity	[7]
22	Notoginsenoside R1	engineered *Saccharomyces cerevisiae*			[8]
23	Notoginsenoside R2	engineered *S. cerevisiae*			[8]

24	Striatin A	*Cyathus striatus*	Mycelium	Antibacterial, antifungal, antileishmanial and antitumor activities	[9-11]
25	Striatin B	*C. striatus*	Mycelium	Antibacterial, antifungal, antileishmanial and antitumor activities	[9-11]
26	Striatin C	*C. striatus*<br>*C. subglobisporus* BCC44381	Mycelium	Antibacterial, antifungal, antileishmanial, antimalarial, anti-tuberculosis and antitumor activities	[9-12]
27	Striatal A	*C. striatus*<br>*C. subglobisporus* BCC44381	Mycelium	Antibacterial, antifungal, antileishmanial, antimalarial, anti-tuberculosis and antitumor activities	[10-13]
28	Striatal B	*C. striatus*	Mycelium	Antibacterial, antifungal, antileishmanial and antitumor activities	[10, 11, 13]
29	Striatal C	*C. striatus*<br>*C. subglobisporus* BCC44381	Mycelium	Antibacterial, antifungal, antileishmanial, antimalarial, anti-tuberculosis and antitumor activities	[10-13]
30	Striatal D	*Gerronema fibula;*<br>*C. striatus;*<br>*C. subglobisporus* BCC44381;<br>*Laxitextum Incrustatum;*<br>*Dentipellis fragilis*	Mycelium	Antibacterial, antifungal, antileishmanial, antimalarial and antitumor activities	[10, 12-15]
31	Striatoid A	*C. striatus*	Mycelium fermentation broth	Neurotrophic activity	[16]
32	Striatoid B	*C. striatus*	Mycelium fermentation broth	Neurotrophic activity	[16]
33	Striatoid C	*C. striatus*<br>*C. subglobisporus* BCC44381	Mycelium fermentation broth	Neurotrophic activity, Antimalarial activity, antibacterial	[12, 16]
34	Striatoid D	*C. striatus*	Mycelium fermentation broth	Neurotrophic activity	[16]
35	Striatoid E	*C. striatus*	Mycelium fermentation broth	Neurotrophic activity	[16]
36	Striatoid F	*C. striatus*	Mycelium	Neurotrophic activity	[16]

			fermentation broth		
37	Cyathinin A	*C. subglobisporus* BCC44381		Antimalarial, antibacterial and anti-tuberculosis activities	[12]
38	Cyathinin B	*C. subglobisporus* BCC44381			[12]
39	Cyathinin C	*C. subglobisporus* BCC44381			[12]
40	Cyathinin D	*C. subglobisporus* BCC44381		Antimalarial, antifungal, antibacterial and anti-tuberculosis activities	[12]
41	Cyathinin E	*C. subglobisporus* BCC44381			[12]
42	10-Hydroxyerinacine S	*C. subglobisporus* BCC44381			[12]
43	Me-dentifragilin A/ Hericinoid B	*C. striatus* CBPFE A06 *Hericium erinaceum*		Neuroprotective and anti-neuroinflammatory activities; Antitumor activity	[17, 18]
44	Erinacine A	*Hericium erinaceum* *H. flagellum* *Dentipellis fragilis*	Mycelium fermentation broth	Neurotrophic activity, Antimicrobial, anti-MRSA and antifungal activities	[19-23]
45	Erinacine B	*H.erinaceum* *H. flagellum* *D. fragilis*	Mycelium fermentation broth	Neurotrophic activity, Antimicrobial and antifungal activities	[19, 22, 23]
46	Erinacine C	*H.erinaceum* *H. flagellum* *D. fragilis*	Mycelium fermentation broth	Neurotrophic activity, Antimicrobial, anti-MRSA and antifungal activities	[19, 21-23]
47	Erinacine D	*H.erinaceum*	Mycelium fermentation broth	Neurotrophic activity	[24, 25]
48	Erinacine E	*H.erinaceum* *H. flagellum*	Mycelium fermentation broth	Neurotrophic activity; Selective antagonist and agonist for the κ receptor	[21, 22, 26, 27]
49	Erinacine F	*H.erinaceum* *H. flagellum*	Mycelium fermentation broth	Neurotrophic activity	[22, 26]
50	Erinacine G	*H.erinaceum*	Mycelium fermentation broth	Neurotrophic activity	[26]
51	Erinacine H	*H.erinaceum*	Mycelium fermentation broth	Neurotrophic activity	[28]
52	Erinacine J	*H.erinaceum*	Mycelium fermentation broth	Neurotrophic activity	[21]

53	Erinacine K	*H.erinaceum*	Mycelium fermentation broth	Neurotrophic activity; Anti-MRSA activity	[21]
54		*H.erinaceum*	Mycelium fermentation broth		[25]
55		*H.erinaceum*	Mycelium fermentation broth		[25]
56	CJ-14,258	*H. ramosum* CL24240 *H. flagellum*		Antagonist for the κ receptor; Neurotrophic activity	[21, 22, 27]
57	CJ-15,544	*H. ramosum* CL24240 *H.erinaceum*		Antagonist for the κ receptor	[18, 27]
58	CP-412,065	*C. fumago* ATCC 16373			[27]
59	Erinacine P	*H. erinaceum* YB4-6237; *H. erinaceum*	Mycelium fermentation broth	Neurotrophic activity	[20, 29]
60	Erinacine Q	*H. erinaceum* YB4-6237	Mycelium fermentation broth		[30]
61	Erinacine R	*H. erinaceum*	Mycelium		[31]
62		*H. erinaceum*	Mycelium	Antibacterial and antitumor activities	[32]
63	Erinacine S	*H. erinaceum*	Mycelium	Anti-Alzheimer's disease	[33]
64	Erinacine T/Z2	*H. erinaceum* *H. flagellum*	Mycelium	Neurotrophic activity; Antitumor activity	[18, 20, 22]
65	Erinacine U/Z1	*H. erinaceum*	Mycelium	Neurotrophic activity; Antitumor activity	[18, 20, 22]
66	Erinacine V	*H. erinaceum*		Neurotrophic activity	[20]
67	Hericinoid A	*H. erinaceum*			[18]
68	Hericinoid C	*H. erinaceum*			[18]
69	Erinacine L	*H. erinaceus* CGMCC 5.579		Neuroprotective and anti-neuroinflammatory activities	[34]
70	Laxitextine A	*Laxitextum incrustatum* *D. fragilis*	Mycelium	Antibacterial, anti-MRSA and antitumor activities	[14]
71	Laxitextine B	*L. incrustatum*	Mycelium	Antibacterial, anti-MRSA and antitumor activities	[14]
72	Dentifragilin A	*Dentipellis fragilis*		Antibacterial and antitumor activities	[15]
73	Dentifragilin B	*D. fragilis*			[15]
74	Dentifragilin C	*D. fragilis*			[15]
75	Dentifragilin D	*D. fragilis*		Antibacterial and antitumor activities	[15]
76	Dentifragilin E	*D. fragilis*		Antibacterial and antitumor activities	[15]
77	Dentifragilin F	*D. fragilis*			[15]
78	Dentifragilin G	*D. fragilis*		Antitumor activity	[15]
79	Dentifragilin H	*D. fragilis*		Antitumor activity	[15]

80	Erinacine W	engineered *S. cerevisiae*		Neurotrophic activity	[35]
81	Erinacine X	engineered *S. cerevisiae*		Neurotrophic activity	[35]
82		engineered *S. cerevisiae*		Neurotrophic activity	[35]
83	Erinacine Y	engineered *S. cerevisiae*		Neurotrophic activity	[35]
84	Erinacine ZA	engineered *S. cerevisiae*		Neurotrophic activity	[35]
85	Erinacine ZB	engineered *S. cerevisiae*			[35]
86	Erinacine ZC	engineered *S. cerevisiae*			[35]
87	Benamomicin A	*Actinomycete* sp. MH193-16F4		Antibacterial, antifungal and anti-AIDS activities	[36, 37]
88	Benamomicin B/ Pradimicin C	*A.* sp. MH193-16F4 *Actinomadura hibisca* P157-2 (ATCC 53557)		Antibacterial, antifungal and anti-AIDS activities	[36-39]
89	2'-demethylbenanomicin A	*A.* sp. MH193-16F4		Antifungal activity	[40]
90	BMY-28567/ Pradimicin A	*Actinomadura hibisca* No. P157-2 (ATCC 53557)		Antibacterial, Antifungal and anti-AIDS activities	[38, 39, 41, 42]
91	Pradimicin D	*A. hibisca* No. P157-2(ATCC 53557)		Antifungal activity	[43]
92	Pradimicin E	*A. hibisca* No. P157-2(ATCC 53557)		Antifungal activity	[43]
93	Pradimicin FA-1	*A. hibisca* No. A2493 (ATCC 53815)		Antifungal activity	[44]
94	Pradimicin FA-2	*A. hibisca* No. A2493 (ATCC 53815)		Antifungal activity	[44]
95	Pradimicin T1	*A.* AA3798		Antifungal and antiviral activities	[45, 46]
96	Pradimicin T2	*A.* AA3798		Antifungal and antiviral activities	[45, 46]
97	Pradimicins H	JN-380			[47]
98	Pradimicins FH	JN-380			[47]
99	11-*O*-L-xylosylpradimicins H	*A.*AA3798		Antifungal activity	[47]
100	11-*O*-L-xylosylpradimicins FH	*A.*AA3798		Antifungal activity	[47]
101	2-methoxy-4-methylphenyl *β*-D-xyloside (MeG-	*Coriolus versicolor*			[48]

	Xyl)				
102	vanillyl *β*-D-xyloside (VA-Xyl-Al)	*C. versicolor*			[48]
103	2 methoxy-4-hydroxymethylphenyl *β*-D-xyloside (VA-Xyl-Ph)	*C. versicolor*			[48]
104		*Trametes versicolor*			[49]
105	masutakeside I	*Laetiporus sulphureus* var. *miniatus*	fruiting bodies	Antitumor antivity	[50]
106	*N*-(4-methoxyphenyl)formamide 2-O-*β*-D-xyloside	*T. versicolor*		Enhances the function of BEAS-2B cell viability	[51]
107	*N*-(4-methoxyphenyl)formamide 2-O-*β*-D-xylobioside	*T. versicolor*			[51]
108	Asterbatanoside A/ Bungeiside C	*Plectosphaerella cucumerina* YCTA2Z1; *Cynanchum bungei* Decne			[52, 53]
109	A-40104A	*Clitopilus pseudo-pinsitus*		Antibacterial activity	[54]
110	Cepacidine A1	*Pseudomonas cepacian* AF 2001		Antifungal activity	[55]
111	Cepacidine A2	*P. cepacian* AF 2001		Antifungal activity	[55]
112	Aeruginosin 205A	*Oscillatoria agardhii* (NIES-205)	Mycelium	Inhibits trypsin and thrombin	[56]
113	Aeruginosin 205B	*O. agardhii* (NIES-205)	Mycelium	Inhibits trypsin and thrombin	[56]
114	Aeruginoside 126A	*Planktothrix agardhii* CYA126/8			[57]
115	Aeruginoside 126B	*P. agardhii* CYA126/8			[57]
116	Occidiofungin A	*Burkholderia contaminans* MS14		Antifungal activity	[58]
117	Occidiofungin B	*B. contaminans* MS14		Antifungal activity	[58]
118	Bk-1229	*B. ambifaria* 2.2N		Antifungal activity	[59]
119	Butirosin A	*Bacillus circulans*		Antibacterial activity	[60]
120	Xylostasin	*B.* sp. Y-399; *B.* sp. V-7		Antibacterial activity	[61]

121	Tjipanazole B	*Tolypothrix tjipanasensis*			[62]
122	Tjipanazole F1	*T. tjipanasensis*			[62]
123	Tjipanazole F2	*T. tjipanasensis*			[62]
124	5-*O*-(α-D-xylopyranosyl) streptazolin	*Streptomyces* sp. strain A1		Antitumor activity	[63]
125	Aleurodiscal	*Aleurodiscus mirabilis*	Mycelium Fermentation broth	Antifungal activity; Cytotoxicity	[64]
126	diapolycopenedioic acid xylosyl ester	*Rubritalea squalenifaciens*		Anti-oxidative activity	[65]

A vacancy in the table indicates that its information is not available.

References

1. Fujimoto H, Suzuki K, Hagiwara H, Yamazaki M. New Toxic Metabolites from a Mushroom, *Hebeloma vinosophyllum*. I. : Structures of Hebevinosides I, II, III, IV, and V. CHEMICAL & PHARMACEUTICAL BULLETIN 1986;34(1):88-99.
2. Fujimoto H, Hagiwara H, Suzuki K, Yamazaki M. New toxic metabolites from a mushroom, *Hebeloma vinosophyllum*. II. Isolation and structures of hebevinosides VI, VII, VIII, IX, X, and XI. Chem Pharm Bull (Tokyo) 1987;35(6):2254-2260.
3. Fujimoto H, Maeda K, Yamazaki M. New Toxic Metabolites from a Mushroom, *Hebeloma vinosophyllum*. III. Isolation and Structures of Three New Glycosides, Hebevinosides XII, XIII and XIV, and Productivity of the Hebevinosides at Three Growth Stages of the Mushroom. CHEMICAL & PHARMACEUTICAL BULLETIN 1991;39(8):1958-1961.
4. Su H-J, Fann Y-F, Chung M-I, Won S-J, Lin C-N. New Lanostanoids of *Ganoderma tsugae.* Journal of Natural Products 2000;63(4):514-516.
5. Yoshikawa K, Matsumoto K, Mine C, Bando S, Arihara S. Five Lanostane Triterpenoids and Three Saponins from the Fruit Body of *Laetiporus versisporus.* CHEMICAL & PHARMACEUTICAL BULLETIN 2000;48(10):1418-1421.
6. Yoshikawa K, Inoue M, Matsumoto Y, Sakakibara C, Miyataka H, Matsumoto H, Arihara S. Lanostane Triterpenoids and Triterpene Glycosides from the Fruit Body of *Fomitopsis pinicola* and Their Inhibitory Activity against COX-1 and COX-2. Journal of Natural Products 2005;68(1):69-73.
7. Peng X-R, Su H-G, Liu J-H, Huang Y-J, Yang X-Z, Li Z-R, et al. C30 and C31 Triterpenoids and Triterpene Sugar Esters with Cytotoxic Activities from Edible Mushroom Fomitopsis pinicola (Sw. Ex Fr.) Krast. Journal of Agricultural and Food Chemistry 2019;67(37):10330-10341.
8. Li X, Wang Y, Fan Z, Wang Y, Wang P, Yan X, Zhou Z. High-level sustainable production of the characteristic protopanaxatriol-type saponins from Panax species in engineered *Saccharomyces cerevisiae.* Metab Eng 2021;66:87-97.
9. Anke T, Oberwinkler F. The striatins--new antibiotics from the basidiomycete *Cyathus striatus* (Huds. ex Pers.) Willd. J Antibiot (Tokyo) 1977;30(3):221-225.
10. Shen T, Hof LM, Hausmann H, Stadler M, Zorn H. Development of an enzyme linked immunosorbent assay for detection of cyathane diterpenoids. BMC Biotechnol 2014;14:98.

11. Hecht H-J, Höfle G, Steglich W, Anke T, Oberwinkler F. Striatin A, B, and C: novel diterpenoid antibiotics from *Cyathus striatus*; X-ray crystal structure of striatin A. Journal of the Chemical Society, Chemical Communications 1978(15):665-666.
12. Nitthithanasilp S, Intaraudom C, Boonyuen N, Suvannakad R, Pittayakhajonwut P. Antimicrobial activity of cyathane derivatives from *Cyathus subglobisporus* BCC44381. Tetrahedron 2018;74(48):6907-6916.
13. Anke T, Rabe U, Schu P, Eizenhöfer T, Schrage M, Steglich W. Studies on the biosynthesis of striatal-type diterpenoids and the biological activity of herical. Z Naturforsch C J Biosci 2002;57(3-4):263-271.
14. M MC, Christian R, Kathrin W, Ali AM, C MJ, Marc S, D SR. Laxitextines A and B, Cyathane Xylosides from the Tropical Fungus *Laxitextum incrustatum.* Journal of natural products 2016;79(4).
15. Chemutai SW, Nico M, Hedda S, Kathrin W, Harald K, Marc S, Clement MJ. Antimicrobial and Cytotoxic Cyathane-Xylosides from Cultures of the Basidiomycete *Dentipellis fragilis.* Antibiotics 2022;11(8):1072.
16. Bai R, Zhang CC, Yin X, Wei J, Gao JM. Striatoids A-F, Cyathane Diterpenoids with Neurotrophic Activity from Cultures of the Fungus *Cyathus striatus.* Journal of Natural Products 2015;78(4):783-788.
17. Wei J, Ye M-Y, Wang Z-X, Zhang Y-L, Hu X-S, Hui H-p, et al. Molecular properties, structure, neurotrophic and anti-inflammatory activities of cultured secondary metabolites from the cultures of the mushroom *Cyathus striatus* CBPFE A06. Natural Product Research 2023:1-6.
18. Chen L, Yao J-N, Chen H-P, Zhao Z-Z, Li Z-H, Feng T, Liu J-K. Hericinoids A–C, cyathane diterpenoids from culture of mushroom *Hericium erinaceus.* Phytochemistry Letters 2018;27:94-100.
19. Kawagishi H, Shimada A, Shirai R, Okamoto K, Ojima F, Sakamoto H, et al. Erinacines A, B and C, strong stimulators of nerve growth factor (NGF)-synthesis, from the mycelia of *Hericium erinaceum.* Tetrahedron Letters 1994;35(10):1569-1572.
20. Zhang Y, Liu L, Bao L, Yang Y, Ma K, Liu H. Three new cyathane diterpenes with neurotrophic activity from the liquid cultures of *Hericium erinaceus.* The Journal of Antibiotics 2018;71(9):818-821.
21. Nakamura TT. Erinacines J and K from the mycelia of *Hericium erinaceum.* Tetrahedron 2006.
22. Rupcic Z, Rascher M, Kanaki S, Köster RW, Stadler M, Wittstein K. Two New Cyathane Diterpenoids from Mycelial Cultures of the Medicinal Mushroom Hericium erinaceus and the Rare Species, Hericium flagellum. International Journal of Molecular Sciences 2018;19(3):740.
23. Ha LS, Ki D-W, Kim J-Y, Choi D-C, Lee I-K, Yun B-S. Dentipellin, a new antibiotic from culture broth of Dentipellis fragilis. The Journal of Antibiotics 2021;74(8):538-541.
24. Kawagishi H, Simada A, Shizuki K, Mori H, Okamoto K, Sakamoto H, Furukawa S. Erinacine D, a stimulator of NGF-synthesis, from the mycelia of *Hericium erinaceum.* Heterocyclic Communications 1996;2(1):51-54.
25. Atsushi S, 篤 島, Hirokazu K, 洋和 河, Shoei F, 昭栄 古, et al., *CYATHANE DERIVATIVE AND INDUCER FOR NERVE GROWTH FACTOR PRODUCTION CONTAINING THE SAME AS ACTIVE INGREDIENT*. 1996.
26. Kawagishi H, Shimada A, Hosokawa S, Mori H, Sakamoto H, Ishiguro Y, et al. Erinacines E, F, and G, stimulators of nerve growth factor (NGF)-synthesis, from the mycelia of *Hericium*

erinaceum. Tetrahedron Letters 1996;37(41):7399-7402.
27. Saito T, Aoki F, Hirai H, Inagaki T, Matsunaga Y, Sakakibara T, et al. Erinacine E as a kappa opioid receptor agonist and its new analogs from a basidiomycete, *Hericium ramosum.* The Journal of antibiotics 1998;51(11):983-90.
28. Lee EW, Shizuki K, Hosokawa S, Suzuki M, Suganuma H, Inakuma T, et al. Two novel diterpenoids, erinacines H and I from the mycelia of *Hericium erinaceum.* Biosci Biotechnol Biochem 2000;64(11):2402-5.
29. Kenmoku H, Sassa T, Kato N. Isolation of erinacine P, a new parental metabolite of cyathane-xylosides, from *Hericium erinaceum* and its biomimetic conversion into erinacines A and B. ChemInform 2000;31(22):4389-4393.
30. Kenmoku H, Shimai T, Toyomasu T, Kato N, Sassa T. Erinacine Q, a New Erinacine from Hericium erinaceum, and its Biosynthetic Route to Erinacine C in the Basidiomycete. Bioscience, Biotechnology, and Biochemistry 2002;66(3):571-575.
31. Bing-Ji, Ma, Yan, Zhou, Lian-Zhen, Li, et al. A New Cyathane-xyloside from the Mycelia of *Hericium erinaceum.* Zeitschrift für Naturforschung B 2008;63(10).
32. Zhang Z, Liu R-N, Tang Q-J, Zhang J-S, Yang Y, Shang X-D. A new diterpene from the fungal mycelia of *Hericium erinaceus.* Phytochemistry Letters 2015;11:151-156.
33. Chen C-C, Tzeng T-T, Chen C-C, Ni C-L, Lee L-Y, Chen W-P, et al. Erinacine S, a Rare Sesterterpene from the Mycelia of *Hericium erinaceus.* Journal of Natural Products 2016;79(2):438-441.
34. Wei J, Li J-y, Feng X-l, Zhang Y, Hu X, Hui H, et al. Unprecedented Neoverrucosane and Cyathane Diterpenoids with Anti-Neuroinflammatory Activity from Cultures of the Culinary-Medicinal Mushroom *Hericium erinaceus.* Molecules 2023;28(17):6380.
35. Ma K, Zhang Y, Guo C, Yang Y, Han J, Yu B, et al. Reconstitution of biosynthetic pathway for mushroom-derived cyathane diterpenes in yeast and generation of new “non-natural” analogues. Acta Pharmaceutica Sinica B 2021;11(09):2945-2956.
36. Takeuchi T, Hara T, Naganawa H, Okada M, Hamada M, Umezawa H, et al. New antifungal antibiotics, benanomicins A and B from an actinomycete. The Journal of antibiotics 1988;41(6):807-811.
37. Hoshino H, Seki J, Takeuchi T. New antifungal antibiotics, benanomicins A and B inhibit infection of T-cell with human immunodeficiency virus (HIV) and syncytium formation by HIV. The Journal of antibiotics 1989;42(2):344-346.
38. Tsunakawa M, Nishio M, Ohkuma H, Tsuno T, Konishi M, Naito T, et al. The structure of pradimicins A, B and C: a novel family of antifungal antibiotics. The Journal of Organic Chemistry 1989;54(11):2532-2536.
39. Oki T, Tenmyo O, Hirano M, Tomatsu K, Kamei H. Pradimicins A, B and C: new antifungal antibiotics. II. In vitro and in vivo biological activities. The Journal of antibiotics 1990;43(7):763-770.
40. Kondo S, Gomi S, Uotani K, Inouye S, Takeuchi T. Isolation of new minor benanomicins. The Journal of antibiotics 1991;44(2):123-129.
41. OKI T, SAITOH K, TOMATSU K, TOMITA K, KONISHI M, KAWAGUCHI H. Novel Antifungal Antibiotic BMY-28567. Annals of the New York Academy of Sciences 1988;544(1):184-187.
42. Tanabe A, Nakashima H, Yoshida O, Yamamoto N, Tenmyo O, Oki T. Inhibitory effect of new

antibiotic, pradimincin A on infectivity, cytopathic effect and replication of human immunodeficiency virus in vitro. The Journal of antibiotics 1988;41 11:1708-1710.

43. Sawada Y, Nishio M, Yamamoto H, Hatori M, Miyaki T, Konishi M, Oki T. New antifungal antibiotics, pradimicins D and E. Glycine analogs of pradimicins A and C. The Journal of antibiotics 1990;43(7):771-777.

44. Sawada Y, Hatori M, Yamamoto H, Nishio M, Miyaki T, Oki T. New antifungal antibiotics pradimicins FA-1 and FA-2: D-serine analogs of pradimicins A and C. The Journal of antibiotics 1990;43(10):1223-1229.

45. Hasegawa T, Kakushima M, Hatori M, Aburaki S, Kakinuma S, Furumai T, Oki T. PRADIMICIN-T1 AND PRADIMICIN-T2, NEW ANTIFUNGAL ANTIBIOTICS PRODUCED BY AN ACTINOMYCETE .2. STRUCTURES AND BIOSYNTHESIS. Journal of Antibiotics 1993;46(4):598-605.

46. Furumai T, Hasegawa T, Kakushima M, Suzuki K, Yamamoto H, Yamamoto S, et al. PRADIMICIN-T1 AND PRADIMICIN-T2, NEW ANTIFUNGAL ANTIBIOTICS PRODUCED BY AN ACTINOMYCETE .1. TAXONOMY, PRODUCTION, ISOLATION, PHYSICOCHEMICAL AND BIOLOGICAL PROPERTIES. Journal of Antibiotics 1993;46(4):589-597.

47. Furumai T, Yamamoto H, Narita Y, Hasegawa T, Aburaki S, Kakushima M, Oki T. MICROBIAL MODIFICATION OF PRADIMICINS AT C-11 LEADING TO 11-O-DEMETHYLPRADIMICINS AND 11-0-L-XYLOSYLPRADIMICINS A AND FA-1. Journal of Antibiotics 1993;46(10):1589-1597.

48. Kondo R, Yamagami H, Sakai K. Xylosylation of Phenolic Hydroxyl Groups of the Monomeric Lignin Model Compounds 4-Methylguaiacol and Vanillyl Alcohol by *Coriolus versicolor*. Appl Environ Microbiol 1993;59(2):438-441.

49. Hundt K, Martin D, Hammer E, Jonas U, Kindermann MK, Schauer F. Transformation of Triclosan by Trametes versicolor and *Pycnoporus cinnabarinus*. Applied and Environmental Microbiology 2000;66(9):4157-4160.

50. Yoshikawa K, Bando S, Arihara S, Matsumura E, Katayama S. A Benzofuran Glycoside and an Acetylenic Acid from the Fungus *Laetiporus sulphureus* var. miniatus. Chemical and Pharmaceutical Bulletin 2001;49(3):327-329.

51. Yao L, Zhu L-P, Xu X-Y, Tan L-L, Sadilek M, Fan H, et al. Discovery of novel xylosides in co-culture of basidiomycetes Trametes versicolor and *Ganoderma applanatum* by integrated metabolomics and bioinformatics. Scientific Reports 2016;6(1):33237.

52. Li J, Kadota S, Kawata Y, Hattori M, Xu GJ, Namba T. Constituents of the roots of *Cynanchum bungei* Decne. Isolation and structures of four new glucosides, bungeiside-A, -B, -C, and -D. Chem Pharm Bull (Tokyo) 1992;40(12):3133-3137.

53. Gu X-J, Ren K, Yao N, Yan S, Zhao J-F, Jiang X-Y, Lian Q. Chemical constituents from endophytic fungus *Plectosphaerella cucumerina* YCTA2Z1 of Cynanchum auriculatum. Chinese Herbal Medicines 2018;10(1):95-98.

54. Michel KH, Higgens CE, *A-40104 antibiotics and process for production thereof*. 1978, Google Patents.

55. Lim Y, Suh JW, Kim S, Hyun B, Kim C, Lee CH. CEPACIDINE-A, A NOVEL ANTIFUNGAL ANTIBIOTIC PRODUCED BY PSEUDOMONAS-CEPACIA .2. PHYSICOCHEMICAL PROPERTIES AND STRUCTURE ELUCIDATION. JOURNAL OF ANTIBIOTICS

1994;47(12):1406-1416.

56. Shin HJ, Matsuda H, Murakami M, Yamaguchi K. Aeruginosins 205A and -B, Serine Protease Inhibitory Glycopeptides from the Cyanobacterium *Oscillatoria agardhii* (NIES-205). The Journal of Organic Chemistry 1997;62(6):1810-1813.
57. Ishida K, Christiansen G, Yoshida WY, Kurmayer R, Welker M, Valls N, et al. Biosynthesis and Structure of Aeruginoside 126A and 126B, Cyanobacterial Peptide Glycosides Bearing a 2-Carboxy-6-Hydroxyoctahydroindole Moiety. Chemistry & Biology 2007;14(5):565-576.
58. Lu S-E, Novak J, Austin FW, Gu G, Ellis D, Kirk M, et al. Occidiofungin, a Unique Antifungal Glycopeptide Produced by a Strain of Burkholderia contaminans. Biochemistry 2009;48(35):8312-8321.
59. Tawfik KA, Jeffs P, Bray B, Dubay G, Falkinham JO, III, Mesbah M, et al. Burkholdines 1097 and 1229, Potent Antifungal Peptides from Burkholderia ambifaria 2.2N. Organic Letters 2010;12(4):664-666.
60. Dion HW, Woo PW, Willmer NE, Kern DL, Onaga J, Fusari SA. Butirosin, a new aminoglycosidic antibiotic complex: isolation and characterization. Antimicrobial agents and chemotherapy 1972;2(2):84-88.
61. Horii S, Nogami I, Mizokami N, Arai Y, Yoneda M. New antibiotic produced by bacteria, 5-beta-D-xylofuranosylneamine. Antimicrobial agents and chemotherapy 1974;5(6):578-581.
62. Bonjouklian R, Smitka TA, Doolin LE, Molloy RM, Debono M, Shaffer SA, et al. Tjipanazoles, new antifungal agents from the blue-green alga *Tolypothrix tjipanasensis.* Tetrahedron 1991;47(37):7739-7750.
63. Puder C, Loya S, Hizi A, Zeeck A. New Co-metabolites of the Streptazolin Pathway. Journal of Natural Products 2001;64(1):42-45.
64. Lauer U, Anke T, Sheldrick WS, Scherer A, Steglich W. Antibiotics from basidiomycetes. XXXI. Aleurodiscal: an antifungal sesterterpenoid from *Aleurodiscus mirabilis* (Berk. & Curt.) Höhn. J Antibiot (Tokyo) 1989;42(6):875-882.
65. Shindo K, Mikami K, Tamesada E, Takaichi S, Adachi K, Misawa N, Maoka T. Diapolycopenedioic acid xylosyl ester, a novel glyco-C30-carotenoic acid produced by a new marine bacterium *Rubritalea squalenifaciens.* Tetrahedron Letters 2007;48(15):2725-2727.
